# *In Vivo* Antihypercholesterolemic Potential of* Swietenia mahagoni* Leaf Extract

**DOI:** 10.1155/2016/2048341

**Published:** 2016-10-13

**Authors:** Naveen Yelaware Puttaswamy, Asna Urooj

**Affiliations:** ^1^Department of Biochemistry, Pooja Bhagavath Memorial Mahajana PG Center, Mysore, India; ^2^Department of Studies in Food Science and Nutrition, University of Mysore, Manasagangothri, Mysore, India

## Abstract

The present investigation aims to evaluate antihypercholesterolemic potential of* Swietenia mahagoni* leaf aqueous extract (MAE) in diet-induced hypercholesterolemic rat model. In the study, Wistar albino rats (170–220 g) were segregated into 5 groups; all the groups except normal control group were given high fat diet to induce hypercholesterolemia. After induction of cholesterolemia, normal control and positive control groups were treated with saline, statin group was treated with atorvastatin, and remaining two groups received MAE in two doses (250 and 500 mg kg^−1^ BW) for a treatment period of one month. After the treatment period, weight of rats was recorded and they were anesthetized and decapitated. Blood samples were taken and triglycerides, total cholesterol, LDL-C, HDL-C, malondialdehyde (MDA), and urea were determined. Liver and kidney were taken for the estimation of lipid peroxides. The positive control group showed higher values of triglycerides (109 ± 5.1 mg/dL), total cholesterol (134 ± 4.6 mg/dL), LDL-C (44 ± 1.2 mg/dL), MDA, and bile acid content when compared to a normal control group (triglycerides (89 ± 3.2 mg/dL), total cholesterol (72 ± 3.4 mg/dL), and LDL-C (28 ± 1.2 mg/dL)). Treatment with MAE decreased the cholesterol levels, HDL-C, ALT, AST, and bilirubin levels and the effect was dependent on the dose. The results of this study indicated that MAE possesses hypolipidemic potential and thus could be useful in the treatment of hypercholesterolemic condition.

## 1. Introduction

Hypercholesterolemia, also known as dyslipidemia, is a pathological condition which is characterized by elevated amounts of cholesterol and lipoproteins in blood plasma [1]. Since the condition may result in various metabolic disorders such as cardiovascular diseases and diabetes, it is one of the major socioeconomic problems and is the result of a combination of a modern lifestyle and dietary habits [2]. On hypercholesterolemic condition, elevated levels of low density lipoproteins are deposited in the subendothelial region of arteries leading to inflammation and building up of plaque ultimately resulting in hypertension, decreased functional potential of metabolic organs (liver and kidney), and also diabetes [3]. Clinical trials conducted on hypercholesterolemic subjects indicate that the drug which reduces the lipid levels has significant positive effects on morbidity and mortality [4]. Improving the elevated levels of LDL cholesterol has been found to reverse the atherosclerosis and hamper progression of cardiovascular diseases [5, 6]. Dyslipidemia is also a common condition associated with uncontrolled type II DM [7].

*Swietenia mahagoni* is a medicinal plant used by folklore as a primary health care need around the globe [8]. The medicinal plant has been widely investigated for various pharmacological effects [9–12]. In our laboratory, the plant has been extensively investigated for its phytochemical composition, antioxidant potential, and antidiabetic potential by various* in vitro* and* in vivo* assays [13, 14]. In our previous study in diabetic animal model we have found that along with glucose lowering ability the extract has potential lipid lowering ability [15]. The present study hypothesis was based on the results of previous experiments. With the background, the study aims to evaluate antihypercholesterolemic potential of* Swietenia mahagoni* leaf aqueous extract (MAE) in the diet-induced hypercholesterolemic rat model.

## 2. Materials and Methods

### 2.1. Chemicals and Reagents

Alanine aminotransferase (ALT), alkaline phosphatase (ALP), aspartate aminotransferase (AST), total protein, albumin, urea, creatinine, total bilirubin, triglycerides, total cholesterol assay kits, and GOD-POD glucose analysis kit were purchased from Aggappe Diagnostics, Ernakulam, India. Reduced glutathione (GSH) and 5,5-dithio(bis)nitrobenzoic acid (DTNB) were purchased from Sigma-Aldrich, Bangalore, India. All the chemicals and reagents used in the study were of analytical grade.

### 2.2. Collection and Preparation of Samples

The leaf of* Swietenia mahagoni* was collected from Mysore district of Karnataka, India, and subsequently identified by Dr. G. R. Shivamurthy, Department of Studies in Botany, University of Mysore, Mysore, India. The collected sample was thoroughly washed under running water to remove adhering dirt and other foreign particles, dried overnight at 50°C, powdered, passed through 60-mesh sieve, and stored in airtight container at 4°C till further use.

### 2.3. Procurements of Animals and Grouping

Healthy male adult rats (*n* = 30) of Wister strain weighing 130–150 g were procured from animal house University of Mysore. The rats were individually housed in polyacrylic cages and maintained at 25 ± 2°C, 45 to 60% RH and 12 h photo period. The rats were acclimatized for this condition on standard pellet diet (Amrut Feeds, Pune, India) and water* ad libitum*. Using random block design protocol, rats were segregated into five different groups as given in Table 1.

### 2.4. Preparation of High Cholesterol Diet (HCD)

High cholesterol diet was prepared mixing the various proportions of the ingredients mentioned in Table 2.

### 2.5. Induction of Hypercholesterolemia

After acclimatization, hypercholesterolemia in the experimental groups (except for normal control group) was induced by feeding high cholesterol diet (HCD) for 4 weeks. Blood was drawn through tail vein, initially and after one month, and cholesterol levels were analyzed in each rat to confirm the onset of hypercholesterolemic condition. Rats with cholesterol levels > 120 mg dL^−1^ were selected for the study.

After induction of hypercholesterolemia in the animals, positive control and normal control groups were treated with saline, standard group was treated with atorvastatin (10 mg Kg^−1^ BW), and two MAE groups were treated with 250 and 500 mg Kg^−1^ BW MAE, respectively, for a period of 6 weeks. During the study, food, water intake, and body weights were determined weekly.

After 6 weeks of the experimental period, the animals were fasted overnight and were euthanized and decapitated at 10 pm (to compensate for the biological rhythm of cholesterol synthesis). The blood was collected by the method of cardiac puncture in procoagulant coated tubes. The blood was allowed to clot by keeping the tubes undisturbed for 2 h. The coagulated blood was centrifuged at 2500 ×g for 20 min to separate the serum. The serum was stored at −20°C until further use. In the serum, total protein, albumin, total cholesterol, triglycerides, HDL, LDL, VLDL cholesterol, bile acids, lipid peroxides, and glutathione were analyzed using diagnostic kits. The VLDL cholesterol was calculated using the relation VLDL cholesterol = TG/5. The activities of AST, ALT, and ALP were determined using respective standard kits, while the activities of antioxidant enzymes SOD and catalase were determined by previously described methods [16, 17].

Liver, kidney, brain, and heart were excised immediately after decapitation, washed with phosphate buffered saline blotted using blotting paper, and weighed. HMG-CoA reductase activity was analyzed in the liver. For homogenization of liver, kidney, and brain, weighed amount of the organs was taken in phosphate buffered saline (1 : 5 w/v) and homogenized in ice-cold condition. The homogenates were centrifuged at 2500 ×g for 5 min to precipitate the unhomogenized components and connective tissue. The supernatant was used for estimation of total protein, glutathione, and TBARS by previously described methods [18, 19].

### 2.6. Statistical Analysis

All the experiments were performed in triplicates. The values for any experiment were mean of the triplicate values. Values are expressed in mean of triplicate experiments with standard deviation. For the evaluation of significant difference, the data were subjected to a one way ANOVA followed by Tukey's multiple comparison test for significant difference at a confidence level of 95% (*P* ≤ 0.05) using SPSS 11.5 software.

## 3. Results

### 3.1. Body Weight

**Figure 1:**
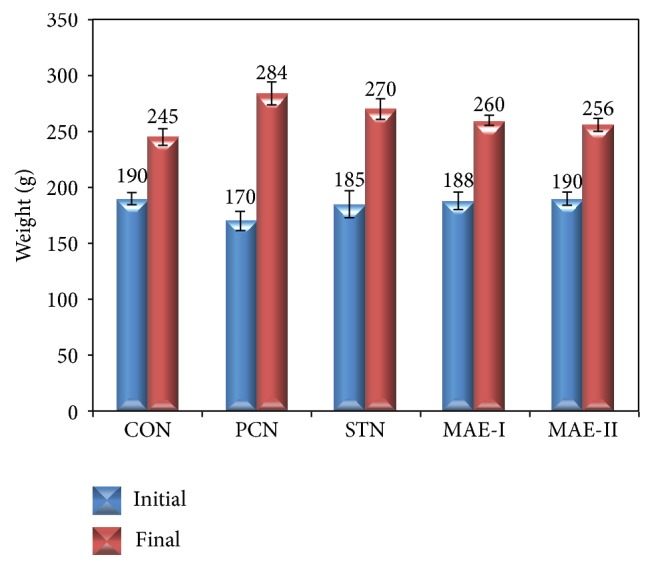
Body weights of experimental groups. CON: control group fed with normal rat chow, PCN: high cholesterol fed positive control group, STN: statin treated high cholesterol fed group, MAE-I: 250 mg kg^−1^ BW mahagoni aqueous extract treated group, and MAE-II: 500 mg kg^−1^ BW mahagoni aqueous extract treated group.

The body weights of experimental groups are presented in Figure 1. It was observed that the increase in the body weight was higher in the experimental groups fed with high cholesterol diet when compared to normal control group fed with normal rodent chow. MAE treatment has reduced the increase in body weight when compared to PCN and STN groups.

### 3.2. Serum Biochemical Parameters

Serum biochemical parameters in the experimental groups are presented in Table 3. It was observed that there were no significant changes in the level of serum protein and albumin was observed between the hypercholesterolemic and normal control groups. However, there was reduction in the levels of urea and creatinine was notable between the hypercholesterolemic and normal control groups. Treatment with MAE has improved the levels of both urea and creatinine towards normal when compared to PCN group and the effect is dose dependent.

### 3.3. Lipid Profile

**Figure 2:**
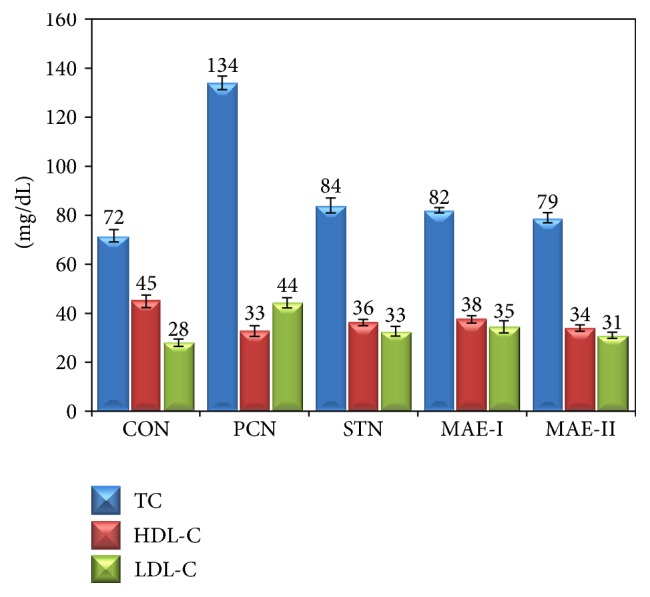
Total cholesterol, HDL, and LDL levels of experimental groups. CON: control group fed with normal rat chow, PCN: high cholesterol fed positive control group, STN: statin treated high cholesterol fed group, MAE-I: 250 mg kg^−1^ BW mahagoni aqueous extract treated group, and MAE-II: 500 mg kg^−1^ BW mahagoni aqueous extract treated group.

**Figure 3:**
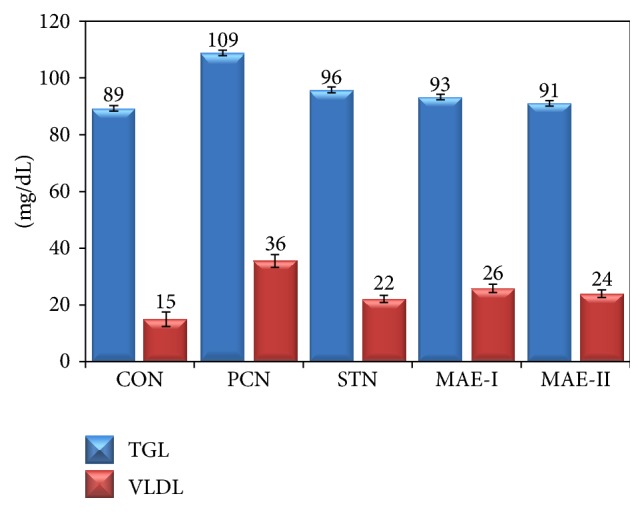
Triglycerides and VLDL levels of experimental groups. CON: control group fed with normal rat chow, PCN: high cholesterol fed positive control group, STN: statin treated high cholesterol fed group, MAE-I: 250 mg kg^−1^ BW mahagoni aqueous extract treated group, and MAE-II: 500 mg kg^−1^ BW mahagoni aqueous extract treated group.

Serum lipid profile of the experimental groups is presented in Figures 2 and 3. There was elevated level of total cholesterol, LDL, VLDL, and triglycerides, whereas there were depleted HDL levels in PCN group when compared to CON group. MAE and STN treatment has improved the levels of HDL and normalized the elevated levels of cholesterol, LDL, and VLDL.

Activity of the enzyme HMG-CoA was measured by measuring amount of CoA released (Table 4). The enzyme activity was highest in PCN group compared to STN, MAE, and CON group. The activity was lowest in MAE treated group and the values were similar to STN group but higher than the CON group. The antioxidant components were reduced in all hyperlipidemic groups compared to CON group. MAE treatment has improved the levels of SOD and catalase as an indication of improvement in the antioxidant potential. Bile acid content was also reduced with MAE treatment; thus, the MAE treatment has improved the antioxidant defense, reduced the activity of HMG-CoA, and reduced the contents of bile acid in the serum.

### 3.4. Lipid Peroxidation in Various Organs

**Figure 4:**
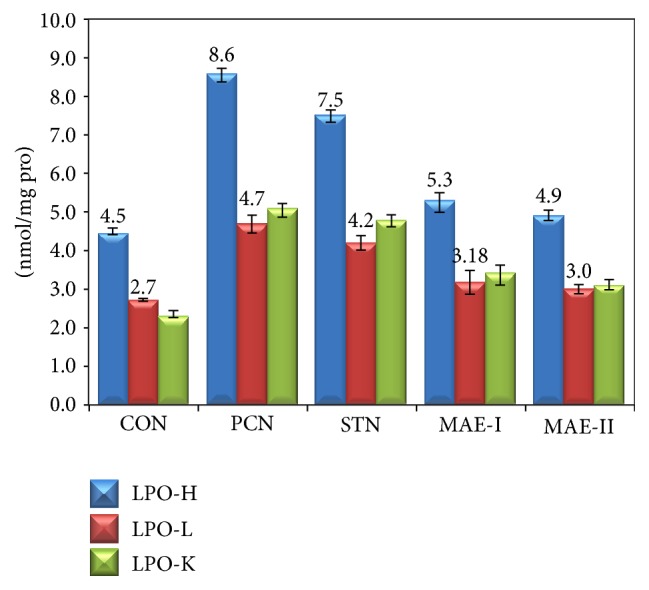
Lipid peroxidation in heart, liver, and kidney of experimental groups. CON: control group fed with normal rat chow, PCN: high cholesterol fed positive control group, STN: statin treated high cholesterol fed group, MAE-I: 250 mg kg^−1^ BW mahagoni aqueous extract treated group, and MAE-II: 500 mg kg^−1^ BW mahagoni aqueous extract treated group.

Lipid peroxidation in heart, liver, and kidney is presented in Figure 4. Lipid peroxide content in all organs was highest in PCN group compared to all experimental groups. Treatment with MAE has reduced the lipid peroxides in all the organs and the reduction was more significant in the STN group and was comparable to CON group.

## 4. Discussion

Feeding of rats with diet containing high content of cholesterol induces hypercholesterolemic condition with impaired lipid profile and antioxidant defense. Oxidative stress, defined as a disruption of the balance between oxidative and antioxidative processes, plays an important role in the pathogenesis of atherosclerosis [20, 21]. Studies in animal models and human clinical trials have established a relationship between hypercholesterolemia and lipid peroxidation [22]. The mechanism of induction of atherogenesis has been shown to be as a result of the accumulation of oxidized lipids within the artery wall. High plasma level of cholesterol, LDL cholesterol in particular, is one of the prominent risk factors for atherosclerosis and thus cardiovascular diseases [23]. Medicinal plants and their derivatives are promisingly gaining wide usage worldwide as they are a potential source of bioactive agents used as pharmaceuticals [24].* Swietenia mahagoni* has a wide usage as pharmaceutical agent in treating diabetes and has anti-inflammatory, antimutagenicity, and antitumour activities [25]. In the present study, there was an elevated level of total cholesterol, LDL, and triglycerides, whereas decreased amounts of HDL and antioxidant enzymes in the PCN group were observed. MAE treatment has improved the antioxidant enzyme levels, reduced the cholesterol content, improved the HDL levels, and reduced the LDL content in the serum, thus promoting the lipid profile and the antioxidant status to a near normal range. The overall phenomenon may be attributed by inhibition of HMG-CoA reductase, which is a regulatory enzyme for the synthesis of cholesterol from the precursors. Statins, a group of hypercholesterolemic drugs, act by inhibiting the HMG-CoA reductase enzyme [26]. Inhibition of cholesterol synthesis further decreases circulating LDL-C because reduced levels of cholesterol in the hepatocyte cause it to upregulate expression of LDL-C receptors. In the present study, there was lower activity of the HMG-CoA in MAE group; the activity was lower than the STN group; the effect may be due to the effect of MAE on the synthesis of the enzyme rather inhibiting its activity.

## 5. Conclusion

With the support of the results of present investigation we conclude that MAE acts to improve the hypercholesterolemic condition by ameliorating dyslipidemia, reducing the content of HMG-CoA, binding of bile acids, and improving the antioxidant status.

## Figures and Tables

**Table 1:** Animal groups, treatment, and dosage (*n* = 6 in each group).

Groups	Treatment	Diet	Dosage
I	CON	Standard diet	—
II	PCN	High cholesterol (2%) diet	—
III	STN	-do-	10 mg kg^−1^ BW
IV	MAE-I	-do-	250 mg kg^−1^ BW
V	MAE-II	-do-	500 mg kg^−1^ BW

CON: healthy control, PCN: untreated positive control, STN: statin, MAE-I: 250 mg kg^−1^ BW mahagoni aqueous extract treated group, and MAE-II: 500 mg kg^−1^ BW mahagoni aqueous extract treated group.

**Table 2:** Composition of the control and high cholesterol diet.

Food ingredients	Control diet (g)	Experimental diet (g)
Wheat flour	62	**60**
Soy flour	18	**18**
Peanut oil	10	**10**
Sugar	7	**7**
Salt mixture	2	**2**
Vitamin mix	1	**1**
Cholesterol	—	**2**
Cholic acid	—	**0.25**

**Table 3:** Effect of samples on serum biochemical parameters of control and diet-induced hypercholesterolemic rats.

Groups	TP	Albumin	Urea	Creatinine
CON	6.42^a^ ± 0.63	4.34^a^ ± 0.38	48.19^b^ ± 7.61	0.64^a^ ± 0.18
PCN	6.92^a^ ± 0.38	3.68^a^ ± 0.19	40.12^a^ ± 2.57	0.74^a^ ± 0.24
STN	6.88^a^ ± 0.42	4.41^a^ ± 0.24	41.34^b^ ± 2.41	0.75^a^ ± 0.42
MAE-I	6.67^a^ ± 0.44	3.98^a^ ± 0.42	43.24^b^ ± 3.18	0.71^a^ ± 0.21
MAE-II	6.52^a^ ± 0.23	3.67^a^ ± 0.34	42.12^b^ ± 2.3	0.69^a^ ± 0.14

CON: control group fed with normal rat chow, PCN: high cholesterol fed positive control group, STN: statin treated high cholesterol fed group, MAE-I: 250 mg kg^−1^ BW mahagoni aqueous extract treated group, and MAE-II: 500 mg kg^−1^ BW mahagoni aqueous extract treated group. Mean values carrying different superscripts a and b in columns differ significantly (*p* ≤ 0.05).

**Table 4:** HMG-CoA reductase activity, bile acid content, and antioxidant enzyme activities of control and diet-induced hypercholesterolemic rats.

Group	HMG-CoA reductase (nM/mg Pro)	Bile acid content (*µ*M/dL)	SOD (IU/mg Pro)	Catalase (IU/mg Pro)
CON	80.0 ± 4.91	0.442 ± 0.06	5.21 ± 1.10	18.83 ± 4.84
PCN	120.0 ± 7.58	0.650 ± 0.02	8.07 ± 2.38	18.43 ± 1.81
STN	62.0 ± 5.60	0.524 ± 0.06	5.08 ± 1.40	20.41 ± 3.68
MAE-I	61.0 ± 4.15	0.398 ± 0.20	4.82 ± 1.25	19.35 ± 3.58
MAE-II	60.15 ± 3.15	0.351 ± 0.18	4.61 ± 0.9	18.5 ± 2.15

CON: control group fed with normal rat chow, PCN: high cholesterol fed positive control group, STN: statin treated high cholesterol fed group, MAE-I: 250 mg kg^−1^ BW mahagoni aqueous extract treated group, and MAE-II: 500 mg kg^−1^ BW mahagoni aqueous extract treated group.

## References

[1] Durrington P. (2003). Dyslipidaemia. *The Lancet*.

[2] Freedman J. E. (2003). High-fat diets and cardiovascular disease: are nutritional supplements useful?. *Journal of the American College of Cardiology*.

[3] Jain K. S., Kulkarni R. R., Jain D. P. (2010). Current drug targets for antihyperlipidemic therapy. *Mini-Reviews in Medicinal Chemistry*.

[4] Levine G. N., Keaney J. F., Vita J. A. (1995). Cholesterol reduction in cardiovascular disease—clinical benefits and possible mechanisms. *The New England Journal of Medicine*.

[5] Ichihashi T., Izawa M., Miyata K., Mizui T., Hirano K., Takagishi Y. (1998). Mechanism of hypocholesterolemic action of S-8921 in rats: S-8921 inhibits ileal bile acid absorption. *Journal of Pharmacology and Experimental Therapeutics*.

[6] Shi W., Haberland M. E., Jien M.-L., Shih D. M., Lusis A. J. (2000). Endothelial responses to oxidized lipoproteins determine genetic susceptibility to atherosclerosis in mice. *Circulation*.

[7] Ginsberg H. N. (2006). Review: efficacy and mechanisms of action of statins in the treatment of diabetic dyslipidemia. *The Journal of Clinical Endocrinology & Metabolism*.

[8] Naveen Y. P. U., Rupini G. D., Ahmed F., Urooj A. (2014). Pharmacological effects and active phytoconstituents of *Swietenia mahagoni*: a review. *Journal of Integrative Medicine*.

[9] Sahgal G., Ramanathan S., Sasidharan S., Mordi M. N., Ismail S., Mansor S. M. (2010). Brine shrimp lethality and acute oral toxicity studies on *Swietenia mahagoni* (Linn.) Jacq. seed methanolic extract. *Pharmacognosy Research*.

[10] Nadi F.-T.-Z. Phytochemical screening, membrane stabilizing activity and cytotoxicity of ethanol extract of swietenia mahagony leaf. http://dspace.bracu.ac.bd/jspui/handle/10361/4662.

[11] Sahgal G., Ramanathan S., Sasidharan S., Mordi M. N., Ismail S., Mansor S. M. (2009). In vitro antioxidant and xanthine oxidase inhibitory activities of methanolic *Swietenia mahagoni* seed extracts. *Molecules*.

[12] Ekimoto H., Irie Y., Araki Y., Han G.-Q., Kadota S., Kikuchi T. (1991). Platelet aggregation inhibitors from the seeds of *Swietenia mahagoni*: inhibition of in vitro and in vivo platelet-activating factor-induced effects of tetranortriterpenoids related to swietenine and swietenolide. *Planta Medica*.

[13] Naveen Y. P., Urooj A. (2015). Phytochemical, proximite composition and antioxidant potential of Swietenia mahagoni leaves. *Asian Journal of Pharmceutical Research*.

[14] Naveen Y. P., Urooj A. (2015). Preclinical safety evaluation of Swietenia Mahagoni leaf in wistar rats. *International Journal of Pharmacy and Pharmaceutical Sciences*.

[15] Naveen Y. P., Urooj A. (2015). Amelioration of diabetes by Swietenia mahagoni in streptozotocin induced diabetic rats. *International Journal of Pharmaceutical Sciences and Research*.

[16] Beauchamp C., Fridovich I. (1971). Superoxide dismutase: improved assays and an assay applicable to acrylamide gels. *Analytical Biochemistry*.

[17] Góth L. (1991). A simple method for determination of serum catalase activity and revision of reference range. *Clinica Chimica Acta*.

[18] Ellman M. (1959). A spectrophotometric method for determination of reduced glutathione in tissues. *Analytical Biochemistry*.

[19] Ohkawa H., Ohishi N., Yagi K. (1979). Assay for lipid peroxides in animal tissues by thiobarbituric acid reaction. *Analytical Biochemistry*.

[20] Rzheshevsky A. V. (2013). Fatal ‘triad’: lipotoxicity, oxidative stress, and phenoptosis. *Biochemistry*.

[21] Cross C. E., van der Vliet A., O'Neill C. A., Louie S., Halliwell B. (1994). Oxidants, antioxidants, and respiratory tract lining fluids. *Environmental Health Perspectives*.

[22] Steinberg D. (2002). Atherogenesis in perspective: hypercholesterolemia and inflammation as partners in crime. *Nature Medicine*.

[23] Tanweer A. J., Chand N., Saddique U., Bailey C. A., Khan R. U. (2014). Antiparasitic effect of wild rue (*Peganum harmala* L.) against experimentally induced coccidiosis in broiler chicks. *Parasitology Research*.

[24] Khoshzaban F., Ghaffarifar F., Koohsari H. R. J. (2014). Peganum harmala aqueous and ethanol extracts effects on lesions caused by *Leishmania major* (MRHO/IR/75/ER) in BALB/c mice. *Jundishapur Journal of Microbiology*.

[25] Guevara A. P., Apilado A., Sakurai H., Kozuka M., Tokuda H. (1996). Anti-inflammatory, antimutagenic and antitumor promoting activities of Mahogany seeds, *Swietenia macrophylla* (Meliaceae). *Philippine Journal of Science*.

[26] Haney E., Carson C., Helfand M., McDonagh M. Drug class review HMG-CoA reductase inhibitors (Statins) and fixed-dose combination products containing a statin. http://s3.amazonaws.com/mwengine2/pubmedhealth/files/pubmedhealth_n_statins09_pdf.pdf.

